# Identifying a target group for selenium supplementation in high-risk cardiac surgery: a secondary analysis of the SUSTAIN CSX trial

**DOI:** 10.1186/s40635-023-00574-8

**Published:** 2023-12-08

**Authors:** Quirin Notz, Daren K. Heyland, Zheng-Yii Lee, Johannes Menger, Johannes Herrmann, Thilo S. Chillon, Stephen Fremes, Siamak Mohammadi, Gunnar Elke, C. David Mazer, Aileen Hill, Markus Velten, Sascha Ott, Maren Kleine-Brueggeney, Patrick Meybohm, Lutz Schomburg, Christian Stoppe

**Affiliations:** 1 Department of Anaesthesiology, Intensive Care, Emergency and Pain Medicine, University Hospital Würzburg, Oberdürrbacher Str. 6, 97080 Würzburg, Germany; 2 Clinical Evaluation Research Unit, Kingston General Hospital, 76 Stuart St, Kingston, ON K7L 2V7 Canada; 3 Department of Critical Care Medicine, Queen’s University, 99 University Ave, Kingston, ON K7L 3N6 Canada; 4 Department of Anaesthesiology, University of Malaya, Lingkungan Budi, 50603 Kuala Lumpur, Malaysia; 5 Department of Cardiac Anaesthesiology and Intensive Care Medicine, Deutsches Herzzentrum der Charité, Augustenburger Platz 1, 13353 Berlin, Germany; 6 Charité Berlin, Institute for Experimental Endocrinology, Hessische Str. 4, 10115 Berlin, Germany; 7 University of Toronto, Sunnybrook Research Institute, 2075 Bayview Ave, Toronto, ON M4N 3M5 Canada; 8 Laval University, Quebec Heart and Lung Institute, 2725 Ch Ste-Foy, Quebec City, QC G1V 4G5 Canada; 9 Department of Anaesthesiology and Intensive Care Medicine, University Medical Center Schleswig-Holstein, Campus Kiel, Arnold-Heller-Str. 3, 24105 Kiel, Germany; 10 St. Michael’s Hospital, Li Ka Shing Knowledge Institute, 38 Shuter St, Toronto, ON M5B 1A6 Canada; 11 Departments of Anesthesiology and Pain Medicine, Physiology and Pharmacology, University of Toronto, 123 Edward Street, Toronto, ON M5G 1E2 Canada; 12 Department of Anaesthesiology and Operative Intensive Care Medicine, University Hospital RWTH Aachen, Pauwelsstr. 30, 52074 Aachen, Germany; 13 Department of Anaesthesiology and Operative Intensive Care Medicine, University Hospital Bonn, Venusberg-Campus 1, 53127 Bonn, Germany; 14 Department of Outcomes Research, Cleveland Clinic, 9500 Euclid Avenue, Cleveland, OH 44195 USA; 15 German Center for Cardiovascular Research, Partner Site Berlin, Potsdamer Str. 58, 10785 Berlin, Germany; 16 Charité-Universitätsmedizin Berlin, Corporate Member of Freie Universität Berlin, Humboldt-Universität zu Berlin, Charitéplatz 1, 10117 Berlin, Germany

**Keywords:** Selenium, Glutathione peroxidase, Cardiac surgery, Critical care, Oxidative stress, SUSTAIN CSX

## Abstract

**Background:**

Recent data from the randomized SUSTAIN CSX trial could not confirm clinical benefits from perioperative selenium treatment in high-risk cardiac surgery patients. Underlying reasons may involve inadequate biosynthesis of glutathione peroxidase (GPx3), which is a key mediator of selenium's antioxidant effects. This secondary analysis aimed to identify patients with an increase in GPx3 activity following selenium treatment. We hypothesize that these responders might benefit from perioperative selenium treatment.

**Methods:**

Patients were selected based on the availability of selenium biomarker information. Four subgroups were defined according to the patient's baseline status, including those with normal kidney function, reduced kidney function, selenium deficiency, and submaximal GPx3 activity.

**Results:**

Two hundred and forty-four patients were included in this analysis. Overall, higher serum concentrations of selenium, selenoprotein P (SELENOP) and GPx3 were correlated with less organ injury. GPx3 activity at baseline was predictive of 6-month survival (AUC 0.73; *p* = 0.03). While selenium treatment elevated serum selenium and SELENOP concentrations but not GPx3 activity in the full patient cohort, subgroup analyses revealed that GPx3 activity increased in patients with reduced kidney function, selenium deficiency and low to moderate GPx3 activity. Clinical outcomes did not vary between selenium treatment and placebo in any of these subgroups, though the study was not powered to conclusively detect differences in outcomes.

**Conclusions:**

The identification of GPx3 responders encourages further refined investigations into the treatment effects of selenium in high-risk cardiac surgery patients.

**Graphical Abstract:**

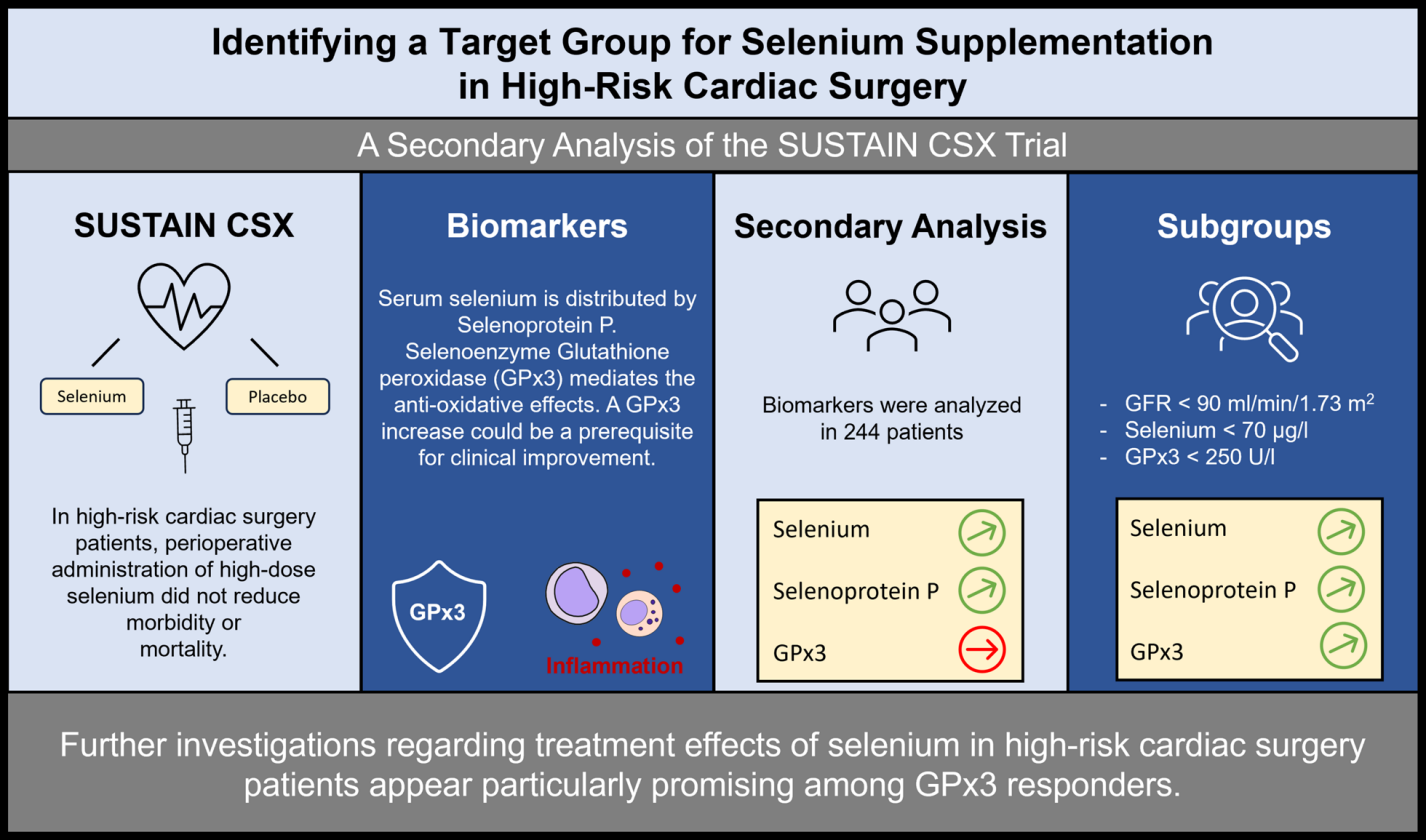

**Supplementary Information:**

The online version contains supplementary material available at 10.1186/s40635-023-00574-8.

## Background

Cardiac surgery provokes a distinct systemic inflammatory response syndrome (SIRS), which has important implications for patients’ mid- and long-term outcomes [1]. Perioperative inflammation and oxidative stress arise from iatrogenic tissue trauma, the use of cardiopulmonary bypass (CPB) and ischemia–reperfusion injury and may represent a modifiable risk factor for the development of organ dysfunction [2–4]. Although it has been a target of research for decades, the complex interactions of pro- and anti-inflammatory cytokines, their determinants, and their influence on the development of SIRS and subsequent organ dysfunction are currently not well understood. In the human body, several endogenous mechanisms specifically protect tissues and organs from reactive oxygen species and their sequelae. The essential trace element selenium is a cornerstone in human antioxidant defense mechanisms due to the pleiotropic anti-inflammatory and immunomodulatory properties of selenoproteins [5–7]. This consideration provides a compelling rationale for selenium treatment in high-risk situations to attenuate the inflammatory response following cardiac surgery and ultimately to improve clinical outcomes. Several observational studies demonstrated a significant intraoperative decrease in selenium levels, and subsequent interventional studies suggested benefits for patients’ short-term outcomes after perioperative selenium supplementation [8, 9]. However, recent data from the multicenter, randomized, placebo-controlled Sodium Selenite Administration in Cardiac Surgery Trial (SUSTAIN CSX) challenged these findings and could not confirm any clinical benefits from intravenous selenium treatment in a broad population of high-risk patients [10]. Furthermore, blood analysis demonstrated that the perioperative provision of high-dose selenium led to a significant increase in serum selenium and selenoprotein P (SELENOP) levels but did not translate to an adequate downstream response, as selenium-dependent plasma glutathione peroxidase (GPx3) remained unaffected [10]. While SELENOP mainly serves as a selenium transporter, kidney-derived GPx3 catalyzes the neutralization of reactive oxygen species and represents the key mediator of the antioxidant capacities of selenium [11–13]. The missing increase in GPx3 activity might therefore explain the absence of measurable clinical effects. Consequently, the objective of this secondary analysis was to identify and characterize subgroups of patients in whom GPx3 activity increases in response to selenium treatment. We hypothesize that these responders might benefit from perioperative selenium treatment.

## Methods

### SUSTAIN CSX overview

This was an a priori defined secondary analysis of the international, double-blind, randomized, placebo-controlled SUSTAIN CSX trial (NCT02002247), which was conducted at 23 centers in Canada and Germany between 2015 and 2021 [10]. Adult patients undergoing elective or urgent cardiac surgery with the use of CPB were eligible if the European System for Cardiac Operative Risk Evaluation II (EuroSCORE II) predicted an operative mortality risk of at least 5% or if combined surgical procedures were scheduled. Patients were randomly assigned to receive either selenium or placebo before surgery and postoperatively throughout the ICU stay [10]. There were no significant differences between the treatment and placebo groups with regard to primary or secondary endpoints, and the results did not identify any clinical benefits of selenium supplementation in high-risk cardiac surgery patients.

### Patient selection and subgroup analyses

Participation in this nested substudy of SUSTAIN CSX was optional for the sites and within the framework of the existing ethical approval [14]. Blood samples were collected, and selenium, SELENOP and GPx3 activity were measured as described [10]. Patients were selected for the current secondary analysis if information on their respective biomarkers was available at any time point. Four subgroups were defined to identify patients who responded to high-dose selenium with an increase in GPx3 activity. These subgroups were as follows:

I. Patients with normal renal function (GFR ≥ 90 ml/min/1.73 m^2^): As the kidneys are the primary source of GPx3, this subgroup may have optimal conditions for an adequate GPx3 response [15].
II. Patients with reduced kidney function (GFR < 90 ml/min/1.73 m^2^): A common finding in organ injury is increased resistance to regulatory stimuli [16]. This resistance might be overcome by high-dose selenium supplementation and result in a GPx3 increase. The threshold of 90 ml/min/1.73 m^2^ has been chosen in accordance with KDIGO guidelines.
III. Patients with selenium deficiency (serum selenium < 70 µg/l): Selenium supplementation might be most effective when serum levels are low and tissues are poorly supplied [7].
IV. Patients with submaximal baseline activity of GPx3 (GPx3 < 250 U/l): In the literature, a threshold of 250 U/l has been established as the upper reference limit for GPx3 to distinguish patients with moderate and high activity. This cutoff value also corresponded to the average GPx3 activity of the present patient population at baseline. By excluding all patients with a priori elevated GPx3 activity from this subgroup, the maximum potential for optimization might be ensured [10, 17].

### Quantification of selenium biomarkers

Patient samples were analyzed at the Institute for Experimental Endocrinology (Charité Berlin, Germany) as recently described [18–20]. Total reflection X-ray fluorescence spectroscopy (S4 T-STAR, Bruker Nano GmbH, Berlin, Germany) was used to determine selenium concentrations. Selenium deficiency was defined as the presence of baseline serum selenium levels < 70 µg/l. For quantification of SELENOP, a commercial enzyme-linked immunosorbent assay kit (selenOtest ELISA, selenOmed GmbH, Berlin, Germany) was used. The activity of GPx3 was assessed via the consumption of nicotinamide adenine dinucleotide phosphate (NADPH) at 340 nm in a coupled enzymatic assay using hydrogen peroxide as a substrate [21]. Reference ranges were used as published elsewhere [22, 23]

### Statistical analyses

The normality of the data was not assumed, and nonparametric testing was applied throughout the manuscript. A *p* value < 0.05 was considered statistically significant (asterisk *), and a *p* value < 0.10 was considered a potential trend (wave ≈). Categorical variables were expressed as absolute numbers and percentages, while continuous variables were presented as the median and interquartile range (IQR, 25–75%). To account for the clustering of sites, categorical outcomes were analyzed by a logistic generalized linear mixed effects model, while continuous outcomes were analyzed by generalized estimating equations. Outcome statistics (Additional file 1: S1, S3–S6) were either reported as odds ratios (ORs), hazard ratios (HRs) or mean differences with 95% confidence intervals (CIs). Mann–Whitney *U* tests were used for further group comparisons.

The correlation coefficient (rho, *r*) quantified associations between continuous variables according to Spearman. To estimate predictive values with regard to survival, receiver operating characteristic (ROC) analyses were conducted and areas under the curves (AUCs) were computed. Statistical analyses were performed using GraphPad Prism^®^ Version 9.5 (GraphPad Software, San Diego, USA) and SAS^®^ Version 9.4 (SAS Institute, Cary, USA).

## Results

### Baseline characteristics and outcomes

**Fig. 1:**
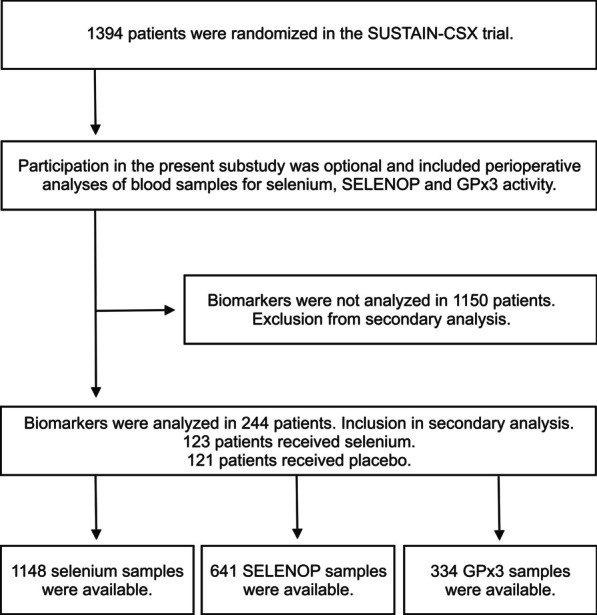
Flow diagram of patient selection. *SELENOP* selenoprotein P, *GPx3* glutathione peroxidase 3

Selenium biomarkers were available in *n* = 244 high-risk cardiac surgery patients who were recruited at 11 participating sites (Fig. 1). Baseline characteristics and outcomes did not differ between the treatment and placebo groups (Table 1; Additional file 1: S1). Five percent of patients died before ICU discharge, and a total of 8% within a 6-month period. Selenium levels at baseline were significantly higher in patients from Canada than in patients from Germany (150, 123–167 µg/l versus 60, 51–74 µg/l; *p* < 0.0001; Additional file 1: S2).

**Table 1:** Demographics

	Selenium (*n* = 123)	Placebo (*n* = 121)	*p*
Baseline characteristics			
Age (years)—median (IQR)	70 (60–76)	71 (64–77)	0.17
Female/male—no. (%)	29 (24)/94 (76)	25 (21)/96 (79)	0.58
Germany/Canada—no. (%)	93 (76)/30 (24)	92 (76)/29 (24)	0.94
Body mass index—median (IQR)	27 (25–30)	28 (25–30)	0.95
Charlson comorbidity index—median (IQR)	1 (0–2)	1 (0–2)	0.41
Clinical frailty score—median (IQR)	3 (2–3)	3 (2–3)	0.34
Baseline chemistry			
GFR (ml/min/1.73 m^2^)—median (IQR)	82 (68–97)	81 (69–97)	0.85
Creatinine (µmol/l)—median (IQR)	84 (73–101)	86 (72–103)	0.94
Interleukin-6 (pg/ml)—median (IQR)	44 (32–49)	36 (22–62)	0.92
Operative characteristics			
EuroSCORE II—median (IQR)	9 (6–16)	8 (6–14)	0.45
Elective/urgent surgery—no. (%)	96 (78)/27 (22)	97 (80) / 24 (20)	0.68
Cardiopulmonary bypass (min)—median (IQR)	140 (113–178)	141 (102–190)	0.90
Baseline micronutrient status			
Selenium (µg/l)—median (IQR)	68 (55—114)	65 (52–119)	0.67
Deficiency (< 70 µg/l)—no. (%)	64 (52)	64 (53)	0.73
SELENOP (mg/l)—median (IQR)	4.5 (3.4–5.7)	4.7 (3.5–6.0)	0.75
GPx3 (U/l)—median (IQR)	273 (187–299)	253 (204–289)	0.62

*GFR* glomerular filtration rate, *GPx3* glutathione peroxidase 3, *IQR* interquartile range, *no.* number of patients, *SELENOP* selenoprotein P

### Perioperative levels of selenium biomarkers

**Fig. 2:**
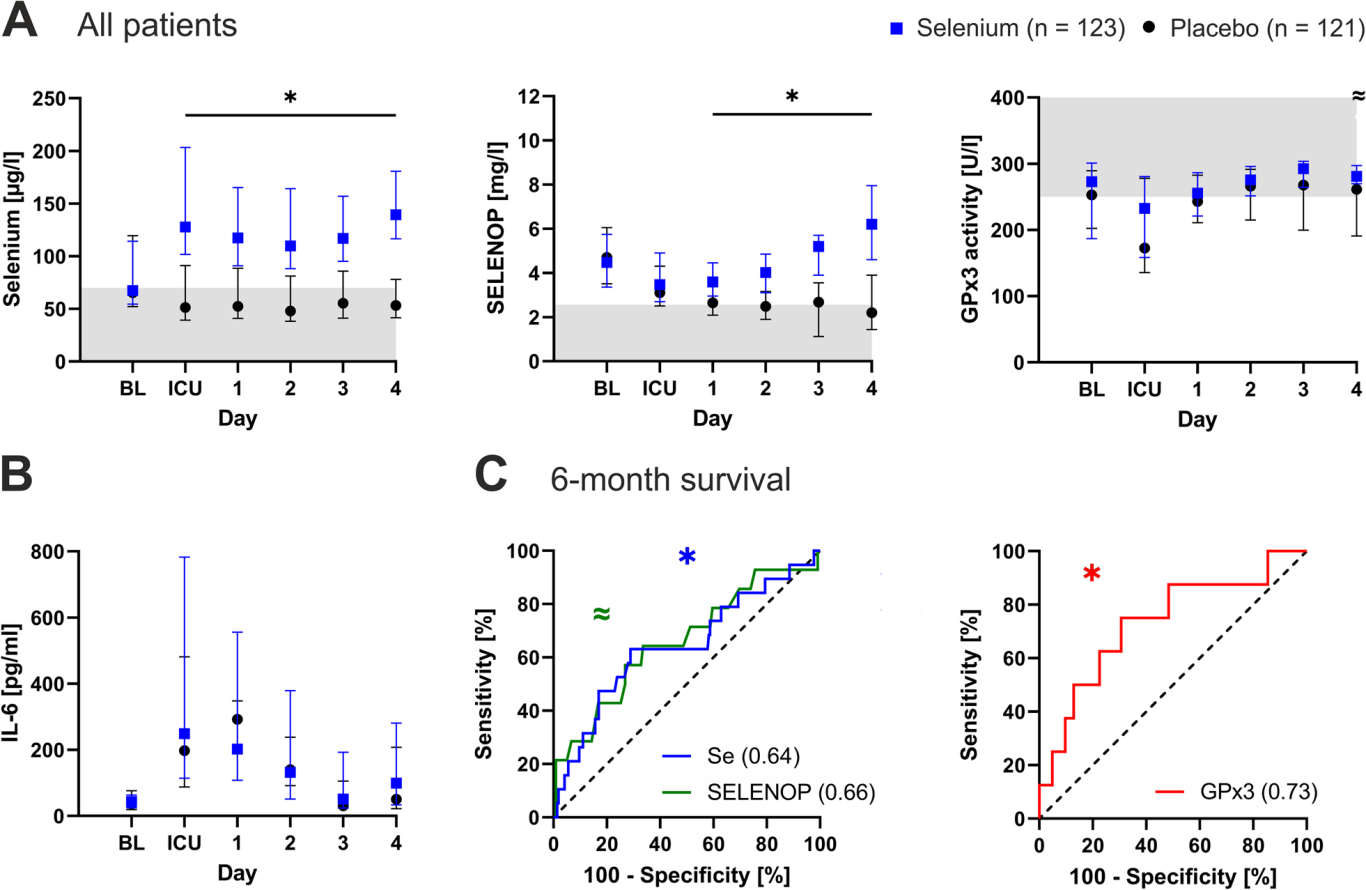
Perioperative selenium status and inflammation. **A** Selenium treatment (blue) increased serum selenium and selenoprotein P (SELENOP) concentrations compared to placebo (black). However, this increase did not translate to altered glutathione peroxidase 3 (GPx3) activity. Gray coloration indicates values below the lower reference limit (selenium and SELENOP) and above the upper reference limit (GPx3). Reference ranges were used as published elsewhere. **B** Interleukin- (IL-) 6 levels reflect a significant inflammatory reaction following cardiac surgery. **C** Receiver operating characteristic (ROC) analyses on the predictive potential of the three selenium biomarkers at baseline regarding 6-month survival after cardiac surgery. Areas under the curves (AUCs) are listed in brackets. GPx3 outperformed the other two biomarkers of selenium status. Asterisks (*) indicate statistical significance, and waves (≈) indicate a trend. *BL* baselinẹ, *ICU* intensive care unit

Baseline serum selenium was below the reference range in half of the patients, while preoperative SELENOP was mostly adequate. Both biomarkers were significantly increased in the treatment group and remained unaffected in patients receiving placebo. The median GPx3 activity was not affected by selenium treatment, as values did not differ between the groups throughout the observation period (Fig. 2A). ICU admission and day one after cardiac surgery were characterized by decreased selenium (placebo group only), SELENOP and GPx3 levels in comparison to baseline and a distinct inflammatory reaction, as reflected by a pronounced spike in interleukin-6 (IL-6) (Fig. 2B). Selenium and SELENOP were closely correlated throughout the study period (e.g., baseline: *r* = 0.70; *p* < 0.0001), whereas GPx3 activity showed only a weak association with selenium (*r* = 0.23; *p* = 0.06) and was not correlated with SELENOP concentrations (*r* = − 0.09; *p* = 0.45).

### Association of selenium biomarkers and clinical outcomes

**Fig. 3:**
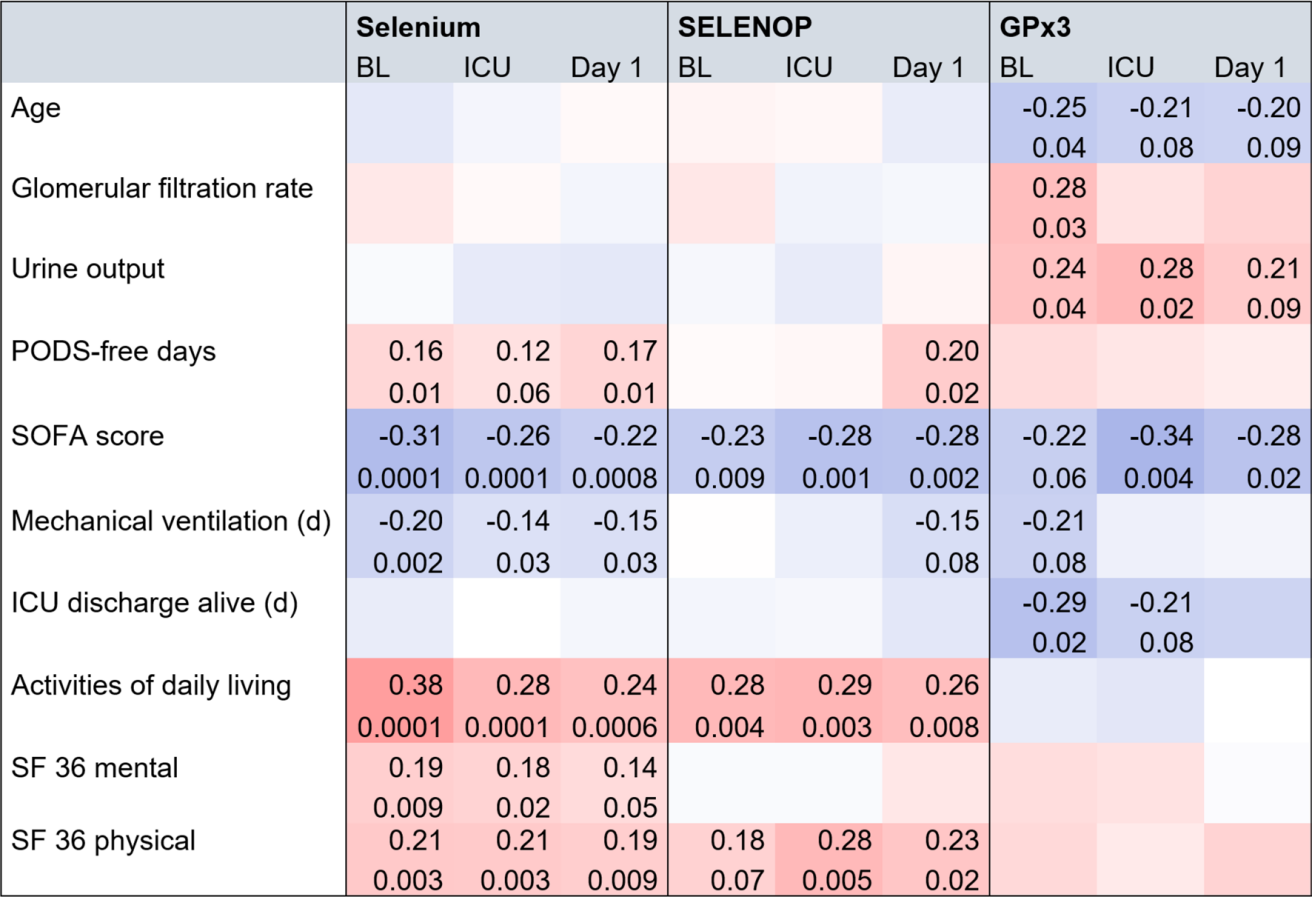
Association between selenium biomarkers and organ injury. Correlation matrix of selenium biomarkers and continuous clinical (outcome) parameters at baseline (BL), admission to the intensive care unit (ICU) and postoperative day one. Positive associations are shown in red, inverse associations in blue. The strength of the relationship is depicted by color graduation. Two values specify each correlation: Spearman’s rho in the upper row and the p value in the lower row. Only significant p values and trends are shown. No relations were observed in the blank fields. The datasets are different in size for each biomarker, with selenium having the most pairs and GPx3 having the fewest. Overall, the associations were weak to moderate. It is important to note that these correlations do not imply causation. *PODS* persistent organ dysfunction and death, *SOFA* sequential organ failure assessment, *SF 36* short-form 36 questionnaire

An explorative correlation analysis revealed multiple significant associations between perioperative selenium biomarkers, clinical status, ICU course and patient-centered outcomes. Overall, higher selenium, SELENOP and GPx3 levels were correlated with less organ injury and a better patient outcome; however, it is important to note that these correlations were of weak and moderate strength, respectively (Fig. 3).

Low GPx3 activity was correlated with older age and reduced kidney function. Low GPx3 activity was also related to a higher score in the sequential organ failure assessment (SOFA), a longer duration of mechanical ventilation and consequently an extended duration to ICU discharge alive.

Regarding long-term outcomes, adequate pre- and postoperative selenium indicated improved functional outcome measures after 3 months. Six-month survivors had higher selenium (67, 55–119 µg/l versus 54, 45–79 µg/l; *p* = 0.04), SELENOP (4.7, 3.5–6.0 mg/l versus 3.7, 2.3–5.3 mg/l; *p* = 0.05) and GPx3 levels (273, 208–296 U/l versus 193, 166–256 U/l; *p* = 0.03) at baseline than nonsurvivors. To assess the predictive potential of baseline biomarkers for 6-month survival after cardiac surgery, ROC curve analyses were performed. All three biomarkers were related to survival, with GPx3 activity (AUC 0.73; *p* = 0.03) outperforming serum selenium (AUC 0.64; *p* = 0.04) and SELENOP (AUC 0.66; *p* = 0.05) concentrations (Fig. 2C).

### GPx3 activity in patients with normal versus impaired kidney function (subgroups I and II)

**Fig. 4:**
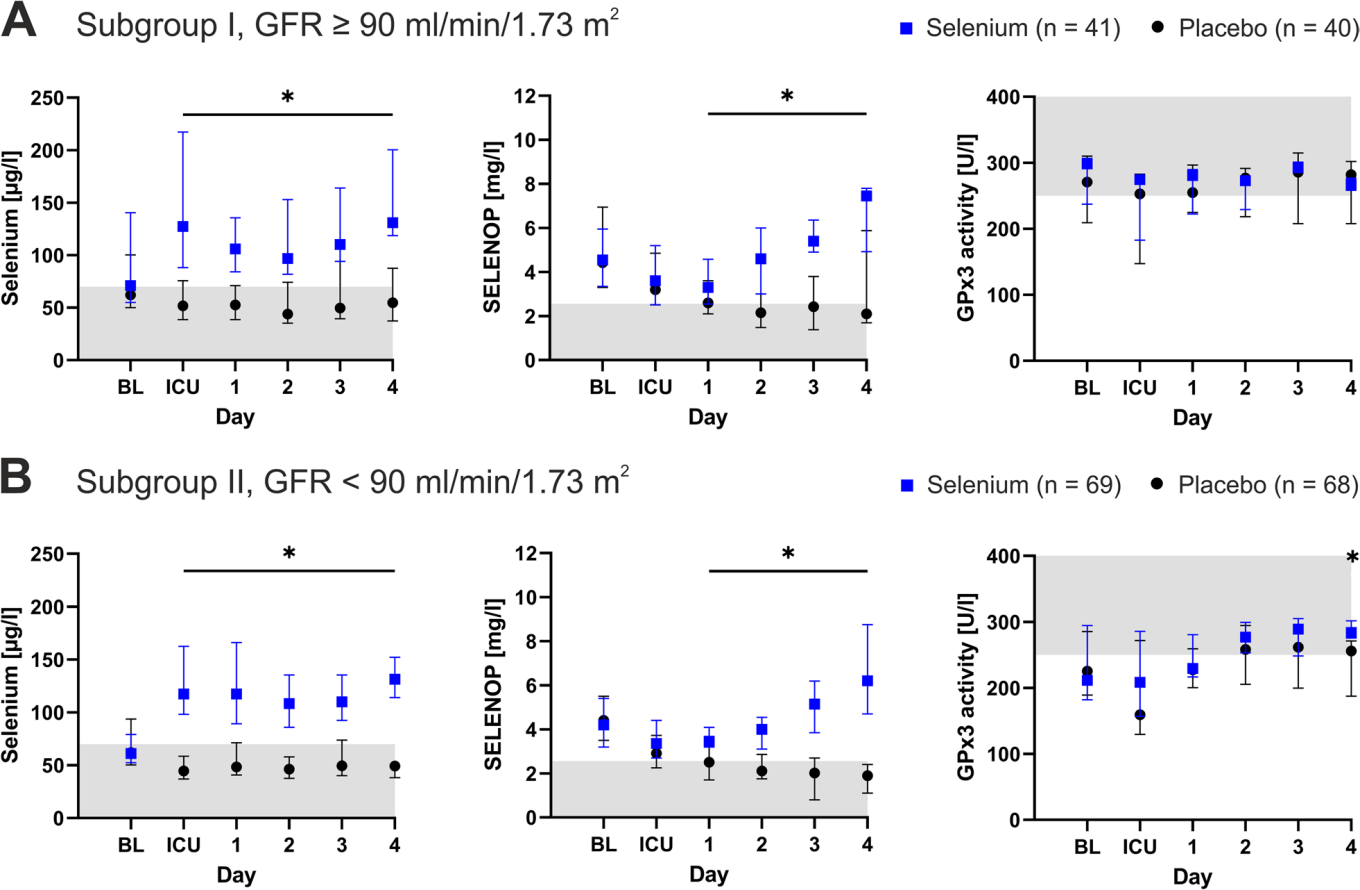
Subgroup I and II. **A** Selenium treatment (blue) increased serum selenium and selenoprotein P (SELENOP) but not glutathione peroxidase 3 (GPx3) activity in patients with normal kidney function according to the KDIGO definition (I). **B** Selenium supplementation in patients with reduced renal function led to an increase in serum selenium, SELENOP and GPx3 at Day 4 (II). Gray coloration indicates values below the lower reference limit (selenium and SELENOP) and above the upper reference limit (GPx3). Reference ranges were used as published elsewhere. Asterisks (*) indicate statistical significance. *BL* baseline, *GFR* glomerular filtration rate, *ICU* intensive care unit

Subgroup I comprised *n* = 81 patients who had a glomerular filtration rate (GFR) of 90 ml/min/1.73 m^2^ or above at baseline. Biomarker levels exhibited the same dynamics as described above, and GPx3 activity did not increase in the treatment group compared to the placebo group (Fig. 4A). Furthermore, no significant outcome differences between the groups were observed (Additional file 1: S3).

Subgroup II included *n* = 137 patients with impaired kidney function and a GFR below 90 ml/min/1.73 m^2^ at baseline. Here, selenium supplementation not only increased serum selenium and SELENOP concentrations, but also GPx3 activity at Day 4 (Fig. 4B). Regarding clinical outcomes in this subgroup, there were no major differences between the treatment and placebo groups. However, the physical domain of the Short Form 36 questionnaire after 3 months tended to be higher with selenium supplementation (46, 41–52 versus 43, 38–51; *p* = 0.05; Additional file 1: S4).

Overall, GPx3 activity was higher in patients with normal kidney function than in patients with renal impairment (291, 214–309 U/l versus 224, 185–290 U/l; *p* = 0.04).

### GPx3 activity in patients with selenium deficiency (subgroup III)

**Fig. 5:**
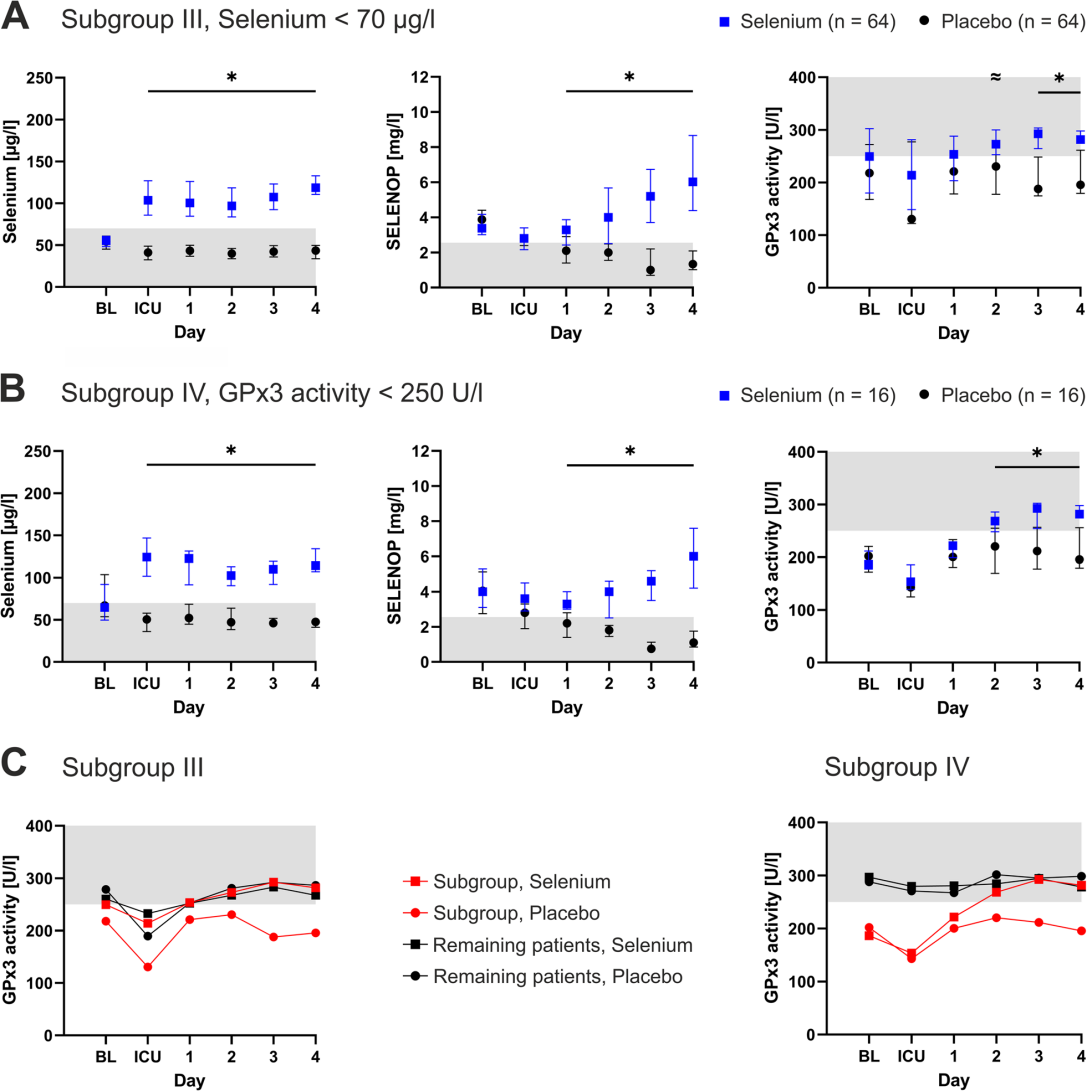
Subgroup III and IV. **A** In patients with selenium deficiency at baseline (defined as values < 70 µg/l), selenium treatment (blue) increased serum selenium, selenoprotein P (SELENOP) and glutathione peroxidase 3 (GPx3) activity (III). **B** In patients with GPx3 activity < 250 U/l at baseline, selenium treatment again successfully increased all biomarkers, including GPx3 (IV). **C** Subgroups III and IV are contrasted with the respective remainder of each patient population. High GPx3 activity remained unchanged throughout the observation period independent of selenium supplementation. For better presentation, error bars were removed, and connecting lines were inserted. Gray coloration indicates values below the lower reference limit (selenium and SELENOP) and above the upper reference limit (GPx3). Reference ranges were used as published elsewhere. Asterisks (*) indicate statistical significance, and waves (≈) indicate a trend. *BL* baseline, *ICU* intensive care unit

Subgroup III comprised all patients with selenium deficiency (< 70 µg/l, *n* = 128) at baseline. Again, selenium treatment elevated serum selenium and SELENOP. In this subgroup, selenium supplementation additionally translated to increased GPx3 activity in comparison to the placebo group starting at postoperative Day 2 (Fig. 5A). However, the evaluated outcomes did not show statistically significant differences between the groups (Additional file 1: S5).

Compared to subgroup III, the patients with preoperative selenium levels ≥ 70 µg/l (*n* = 109) also had higher GPx3 activity at baseline (279, 211–297 U/l versus 226, 178–293; p = 0.05). Here, GPx3 did not differ between patients receiving selenium supplementation and patients receiving placebo throughout the course of intensive care (Fig. 5C).

### GPx3 activity in patients with submaximal baseline GPx3 levels (subgroup IV)

Subgroup IV consisted of patients with GPx3 baseline activity below 250 U/l (*n* = 32). Selenium treatment triggered an adequate biological response, including an increase in serum selenium, SELENOP and GPx3 (Fig. 5B). GPx3 optimization was achieved until Day 2, when a stable plateau was reached. Similarly, the GPx3 activity of patients receiving placebo remained below 250 U/l throughout the observation period. Again, the outcome analysis did not reveal significant differences between the groups (Additional file 1: S6).

In contrast to subgroup IV, the patients with baseline GPx3 activity ≥ 250 U/l (*n* = 38) were not affected by selenium supplementation. Their GPx3 activity constantly remained unchanged at the plateau level (Fig. 5C).

## Discussion

The SUSTAIN CSX intervention trial is currently the most comprehensive study on selenium supplementation in high-risk cardiac surgery patients [10].

Consistent with the original report, intravenous selenium supplementation elevated serum selenium and SELENOP concentrations in the present secondary analysis. The increase of SELENOP ensured efficient uptake and metabolism of the supplemental trace element in the liver and further indicated an improved transport and distribution capacity of selenium throughout the body [24, 25]. Surprisingly, GPx3 failed to display a similar positive response to selenium supplementation and enhanced SELENOP status, with clinical outcomes being similar between the intervention and placebo groups. Closer investigation revealed a transient decline in all selenium biomarkers and a simultaneous peak in inflammatory IL-6 upon surgery, which may indicate the consumption of antioxidant capacity during and after cardiac surgery [9, 26]. Not only did selenium and especially GPx3 activity correlate with kidney function, organ dysfunction and ICU length of stay, but GPx3 activity also emerged as a good predictive marker for long-term survival. This is in line with a recent study, that proposed an early GPx3-based prediction model for acute kidney injury after cardiac surgery [27].

Several subgroups were studied in this secondary investigation to facilitate the identification of a particular responder phenotype of high-risk cardiac surgery patients.

First, selenium biomarkers were analyzed with regard to current renal function, as the renal proximal convoluted tubules are the primary source of GPx3 (Subgroups I and II). As expected, GPx3 correlated with GFR and urine output and was low in patients with impaired kidney function. While selenium supplementation did not lead to increased GPx3 production in patients with normal kidney function, it gradually elevated GPx3 in patients with reduced kidney function. In this subgroup, a trend toward a better functional status after 3 months was observed for selenium treatment in comparison to placebo. While the exact reasons for these findings are unclear, chronic disease is often accompanied by increased resistance to finely adjusted regulatory pathways (e.g., treatment-resistant hypertension in kidney disease, insulin-resistance in metabolic syndrome) [28]. It seems conceivable that chronic renal impairment may elevate the threshold to induce a GPx3 response and that high-dose selenium supplementation could sufficiently provide the requisite signal in comparison to placebo.

Next, supplementation was analyzed in the context of selenium deficiency (Subgroup III). For ICU patients, strong correlations between selenium and GPx3, as well as favorable effects of selenium supplementation have been established [29–33]. Those patients were usually characterized by significantly decreased selenium levels due to SIRS and multiorgan failure [34]. In a previous supplementation trial in critical illness, Angstwurm et al. observed a significant GPx3 increase following selenium treatment in patients with sepsis and septic shock [35]. In line with these results, the present subgroup of patients with preoperative selenium deficiency exhibited an adequate treatment response to selenium supplementation. Nevertheless, clinical outcomes did not differ between the groups, despite previous data considering low levels of selenium as a relevant preoperative risk factor [36, 37].

Finally, the potential for GPx3 optimization was considered the largest in patients with low and moderate GPx3 activity at baseline (Subgroup IV). In healthy subjects, circulating SELENOP and GPx3 exhibited saturation kinetics as a function of serum selenium levels [38–40]. Similarly, a plateau of GPx3 activity was observed in the present cardiac surgery population. This might constitute a biological limit under perioperative conditions, where a surplus of selenium could not be translated into more antioxidant activity. Consequently, patients with above-average GPx3 activity at baseline might only have a small biological margin for improvement. In agreement with this hypothesis, selenium supplementation in Subgroup IV led to a significant increase in SELENOP and GPx3 activity. However, outcomes again did not differ between selenium treatment and placebo.

In summary, subgroup analyses revealed that patients with GFR below 90 ml/min/1.73 m^2^, selenium below 70 µg/l and GPx3 activity below 250 U/l at baseline were likely to respond to selenium supplementation with an increase in GPx3 activity, which may be a prerequisite for clinical improvement. A common property of all three subgroups was submaximal GPx3 activity at baseline, which can therefore be considered a treatable trait. This concept refers to a clinically important patient characteristic with direct physiological implications and a targeted approach for individualized treatment intervention [41, 42]. In the future, preoperative GPx3 measurement as well as the identification of specific risk constellations, including factors such as advanced age and the knowledge of the above defined subgroups, may help to improve the precision and success of adjuvant high-dose selenium treatment.

Despite these promising findings, the clinical relevance of adequate biological downstream translation could not be shown in the present study. Correlation analyses do not allow conclusions about causation, and outcome differences between GPx3 responders and nonresponders were not found. On the one hand, our dataset was not powered to reliably detect outcome differences. On the other hand, in vitro and clinical data, as well as the present observations, suggest that at least a few days of selenium administration are required to stimulate a significant increase in GPx3 activity [9, 43]. One could therefore argue that outcome-relevant GPx3 optimization could not be achieved due to the dosing regimen of the SUSTAIN CSX trial: the first selenium dose was administered shortly prior to CPB and the inflammatory insult, leaving little time for selenoprotein biosynthesis and tissue protection. In fact, a previous study suggested that early initiation of micronutrient supplementation several weeks before elective cardiac surgery improved redox status and shortened the length of hospital stay [44].

Other limitations of the current secondary analysis include the small number of participating patients and the limited amount of biomarker datapoints, which render our investigation strictly hypothesis generating. For this reason, a subgroup analysis of patients with severe selenium deficiency below 45 µg/l could not be performed, despite being highly relevant. In addition, GPx3 regulatory mechanisms are far more complex than selenium status alone, including peroxisome proliferator-activated receptor signaling and hypoxia-inducible as well as other transcription factors [45]. In this context, a definite GPx3 target for optimal organ protection has not been established yet. In this study, the hierarchy of selenoproteins was simplified, as GPx3 is only one member of a large family of selenium-dependent enzymes and GPx isoforms [13]. However, it can be argued that SELENOP and GPx3, as the only actively secreted extracellular selenoproteins, account for the majority of circulating selenium in the serum and bear the most clinical relevance [17, 45].

## Conclusions

A GPx3 increase in response to selenium treatment was observed in patients with GFR below 90 ml/min/1.73 m^2^, selenium below 70 µg/l and GPx3 activity below 250 U/l at baseline. These characteristics might constitute a specific responder phenotype, where further investigations regarding a potential positive treatment effect of selenium supplementation seem promising.

## Take-home message

This secondary analysis of the SUSTAIN CSX trial identified different subgroups of GPx3 responders following selenium treatment. The baseline characteristics of these patients included impaired renal function, serum selenium deficiency and submaximal GPx3 activity. Although clinical differences between selenium treatment and placebo could not be observed in any subgroup, further studies may build on these findings to define a specific responder phenotype that may be susceptible to anti-inflammatory treatment strategies with high-dose selenium.

### Supplementary Information

**Additional file 1**: **S1**: Outcomes in all patients. **S2**: Baseline selenium levels in Canada and Germany. **S3**: Baseline characteristics and outcomes in patients with baseline glomerular filtration rate (GFR) ≥ 90 ml/min/1.73 m^2^. **S4**: Baseline characteristics and outcomes in patients with baseline glomerular filtration rate (GFR) < 90 ml/min/1.73 m^2^. **S5**: Baseline characteristics and outcomes in patients with selenium baseline levels < 70 µg/l. **S6**: Baseline characteristics and outcomes in patients with glutathione peroxidase (GPx3) baseline activity < 250 µg/l.

## Data Availability

The datasets used and analyzed during the current study are available from the corresponding author on reasonable request.

## Abbreviations

BL: Baseline
GFR: Glomerular filtration rate
GPx3: Glutathione peroxidase 3
ICU: Intensive care unit
IL: Interleukin
PODS: Persistent organ dysfunction and death
SELENOP: Selenoprotein P
SF 36: Short-form 36 Questionnaire
SOFA: Sequential Organ Failure Assessment
