# Supplementary material for: Identifying a target group for selenium supplementation in high-risk cardiac surgery: a secondary analysis of the SUSTAIN CSX trial
Source: Intensive Care Med Exp. 2023 Dec 8;11:89. doi: 10.1186/s40635-023-00574-8 (PMC10709283; doi:10.1186/s40635-023-00574-8)
Supplement: Supplementary file 1 — Additional file 1: S1: Outcomes in all patients. S2: Baseline selenium levels in Canada and Germany. S3: Baseline characteristics and outcomes in patients with baseline glomerular filtration rate (GFR) ≥ 90 ml/min/1.73 m2. S4: Baseline characteristics and outcomes in patients with baseline glomerular filtration rate (GFR) < 90 ml/min/1.73 m2. S5: Baseline characteristics and outcomes in patients with selenium baseline levels < 70 µg/l. S6: Baseline characteristics and outcomes in patients with glutathione peroxidase (GPx3) baseline activity < 250 µg/l. [file 40635_2023_574_MOESM1_ESM.docx]

**Additional file 1**

***S1: Outcomes in all patients. - 2***

***S2: Baseline selenium levels in Canada and Germany. - 3***

***S3: Baseline characteristics and outcomes in patients with baseline glomerular filtration rate (GFR) ≥ 90 ml/min/1.73 m^2^. - 4***

***S4: Baseline characteristics and outcomes in patients with baseline glomerular filtration rate (GFR) < 90 ml/min/1.73 m^2^. - 6***

***S5: Baseline characteristics and outcomes in patients with selenium baseline levels < 70 µg/l. - 8***

***S6: Baseline characteristics and outcomes in patients with glutathione peroxidase*** ***(GPx3) baseline activity < 250 µg/l. - 10***

***S1: Outcomes in all patients.***

| **All patients** | **Selenium (n = 123)** | **Placebo (n = 121)** | **OR, HR or mean differences (95 % CI)** | **p value** |
| --- | --- | --- | --- | --- |
| **Primary outcome** |  |  |  |  |
| Persistent organ dysfunction free days and alive at postoperative day 30 (d) - median (IQR) | 29 (28 - 30) | 29 (28 - 30) | -0.3 (-1.7 - 1.2) | 0.75 |
| **Secondary outcomes** | | | | |
| 30-day mortality - no. (%) | 4 (3) | 6 (5) | 0.6 (0.2 - 2.4) | 0.51 |
| 6-months mortality - no. (%) | 10 (8) | 10 (8) | 1.0 (0.4 - 2.5) | 0.98 |
| Days to discharge alive from ICU (d) - median (IQR) | 2 (1 - 6) | 2 (1 - 5) | 0.9 (-1.2 - 2.9) | 0.44 |
| ICU readmission - no. (%) | 3 (2) | 6 (5) | 0.5 (0.1 - 2.0) | 0.31 |
| Mechanical ventilation (d) - median (IQR) | 0 (0 -1) | 0 (0 - 1) | 0.2 (-0.6 - 1.0) | 0.62 |
| Patients with hospital-acquired infections - no. (%) | 13 (11) | 12 (10) | 1.1 (0.5 - 2.4) | 0.90 |
| Barthel Index 3 months - median (IQR) | 100 (100 - 100) | 100 (100 - 100) | 0.9 (-1.3 - 3.1) | 0.42 |
| SF 36 mental 3 months - median (IQR) | 50 (39 - 57) | 50 (40 - 57) | 0.6 (-3.8 - 4.9) | 0.80 |
| SF 36 physical 3 months - median (IQR) | 46 (40 - 52) | 46 (39 - 52) | 0.9 (-1.0 - 2.8) | 0.40 |
| 6-minute walking test at hospital discharge (m) – median (IQR) | 240 (142 - 344) | 240 (160 - 340) | -1.0 (-56.0 - 53.9) | 0.97 |

*CI, confidence interval; HR, hazard ratio; ICU, intensive care unit; IQR, interquartile range; no., number of patients; OR, odds ratio; SF 36, short form questionnaire 36. Outcome analyses are purely explorative, as the dataset is not powered to reliably detect differences between the groups.*


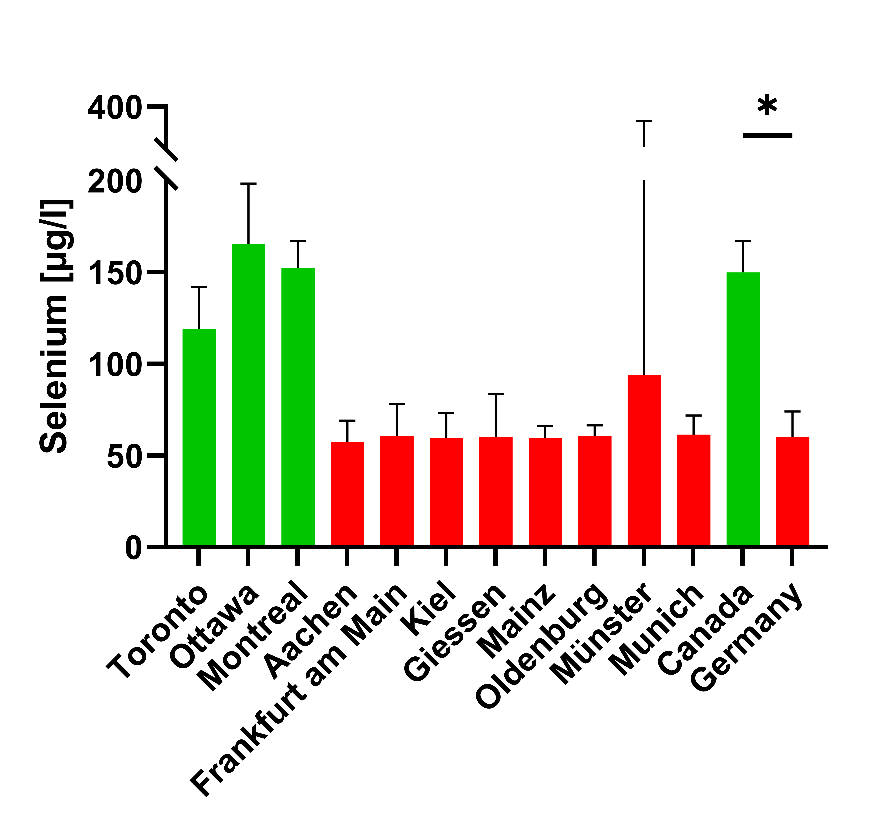


***S2:*** ***Baseline selenium levels in Canada and Germany.*** *Patients from Canada (green) had significantly (*) higher preoperative selenium levels compared to patients from Germany (red; p < 0.0001).*

***S3: Baseline characteristics and outcomes in patients with baseline glomerular filtration rate (GFR) ≥ 90 ml/min/1.73 m^2^.***

| **Baseline GFR ≥ 90 ml/min/1.73 m^2^ (Subgroup I)** | **Selenium**  **(n = 41)** | **Placebo**  **(n = 40)** | **p value** |
| --- | --- | --- | --- |
| **Baseline characteristics** | | |  |
| Age (years) - median (IQR) | 61 (56 - 71) | 64 (59 - 71) | 0.48 |
| Female / Male - no. (%) | 7 (17) / 34 (83) | 5 (13) / 35 (87) | 0.56 |
| Body mass index - median (IQR) | 28 (26 - 31) | 29 (25 - 33) | 0.47 |
| Charlson comorbidity index - median (IQR) | 1 (0 - 1) | 1 (0 - 2) | 0.88 |
| Clinical frailty score - median (IQR) | 3 (2 - 3) | 3 (2 - 3) | 0.94 |
| EuroSCORE II - median (IQR) | 8 (6 - 16) | 8 (5 - 11) | 0.13 |
| Elective / Urgent surgery - no. (%) | 36 (88) / 5 (12) | 36 (90) / 4 (10) | 0.75 |
| Cardiopulmonary bypass (min) - median (IQR) | 143 (120 - 180) | 141 (98 - 177) | 0.51 |
| GFR (ml/min/1.73 m^2^) - median (IQR) | 103 (96 - 114) | 102 (94 - 117) | 0.90 |

*GFR, glomerular filtration rate; IQR, interquartile range; no., number of patients.*

| **Baseline GFR ≥ 90 ml/min/1.73 m^2^ (Subgroup I)** | **Selenium (n = 41)** | **Placebo (n = 40)** | **OR, HR or mean differences (95 % CI)** | **p value** |
| --- | --- | --- | --- | --- |
| **Primary outcome** |  |  |  |  |
| Persistent organ dysfunction free days and alive at postoperative day 30 (d) - median (IQR) | 29 (28 - 30) | 29 (29 - 30) | 0.0 (-2.2 - 2.1) | 0.97 |
| **Secondary outcomes** | | | | |
| 30-day mortality - no. (%) | 1 (2) | 1 (3) | 1.0 (0.1 – 17.0) | 0.98 |
| 6-months mortality - no. (%) | 1 (2) | 1 (3) | 1.0 (0.1 – 17.0) | 0.98 |
| Days to discharge alive from ICU (d) - median (IQR) | 2 (1 - 4) | 2 (1 - 4) | 0.5 (-0.8 - 1.7) | 0.52 |
| ICU readmission - no. (%) | 1 (2) | 1 (3) | 1.0 (0.1 – 17.0) | 0.99 |
| Mechanical ventilation (d) - median (IQR) | 0 (0 - 1) | 0 (0 - 1) | -0.3 (-1.2 - 0.5) | 0.47 |
| Patients with hospital-acquired infections - no. (%) | 4 (10) | 2 (5) | 2.1 (0.3 - 13.4) | 0.43 |
| Barthel Index 3 months - median (IQR) | 100 (100 - 100) | 100 (100 - 100) | 2.7 (-2.3 - 7.7) | 0.30 |
| SF 36 mental 3 months - median (IQR) | 52 (37 - 56) | 51 (35 - 57) | -1.0 (-6.9 - 4.9) | 0.76 |
| SF 36 physical 3 months - median (IQR) | 46 (38 - 54) | 47 (41 - 53) | -0.4 (-4.7 - 3.9) | 0.85 |
| 6-minute walking test at hospital discharge (m) – median (IQR) | 252 (173 - 405) | 240 (190 - 333) | 28.2 (-49.5 - 105.8) | 0.42 |

*CI, confidence interval; HR, hazard ratio; ICU, intensive care unit; IQR, interquartile range; no., number of patients; OR, odds ratio; SF 36, short form questionnaire 36. Outcome analyses are purely explorative, as the dataset is not powered to reliably detect differences between the groups.*

***S4: Baseline characteristics and outcomes in patients with baseline glomerular filtration rate (GFR) < 90 ml/min/1.73 m^2^.***

| **Baseline GFR < 90 ml/min/1.73 m^2^ (Subgroup II)** | **Selenium**  **(n = 69)** | **Placebo**  **(n = 68)** | **p value** |
| --- | --- | --- | --- |
| **Baseline characteristics** | | |  |
| Age (years) - median (IQR) | 72 (66 - 77) | 75 (68 - 78) | 0.14 |
| Female / Male - no. (%) | 20 (29) / 49 (71) | 18 (27) / 50 (73) | 0.74 |
| Body mass index - median (IQR) | 26 (25 - 29) | 27 (24 - 29) | 0.98 |
| Charlson comorbidity index - median (IQR) | 2 (0 - 2) | 2 (0 - 3) | 0.23 |
| Clinical frailty score - median (IQR) | 3 (2 - 4) | 3 (2 - 4) | 0.25 |
| EuroSCORE II - median (IQR) | 11 (7 - 17) | 9 (7 - 19) | 0.97 |
| Elective / Urgent surgery - no. (%) | 55 (80) / 14 (20) | 56 (82) / 12 (18) | 0.69 |
| Cardiopulmonary bypass (min) - median (IQR) | 134 (111 - 167) | 135 (100 - 190) | 0.61 |
| GFR (ml/min/1.73 m^2^) - median (IQR) | 72 (59 - 79) | 73 (62 – 80) | 0.62 |

*GFR, glomerular filtration rate; IQR, interquartile range; no., number of patients.*

| **Baseline GFR < 90 ml/min/1.73 m^2^ (Subgroup II)** | **Selenium (n = 69)** | **Placebo (n = 68)** | **OR, HR or mean differences (95 % CI)** | **p value** |
| --- | --- | --- | --- | --- |
| **Primary outcome** |  |  |  |  |
| Persistent organ dysfunction free days and alive at postoperative day 30 (d) - median (IQR) | 29 (27 - 30) | 29 (27 - 30) | -0.3 (-2.4 - 1.9) | 0.81 |
| **Secondary outcomes** | | | | |
| 30-day mortality - no. (%) | 3 (4) | 5 (7) | 0.6 (0.1 - 2.6) | 0.46 |
| 6-months mortality - no. (%) | 7 (10) | 9 (13) | 0.8 (0.3 - 2.2) | 0.62 |
| Days to discharge alive from ICU (d) - median (IQR) | 3 (1 - 8) | 3 (1 - 5) | 1.1 (-2.4 - 4.5) | 0.57 |
| ICU readmission - no. (%) | 2 (3) | 4 (6) | 0.5 (0.1 - 2.8) | 0.41 |
| Mechanical ventilation (d) - median (IQR) | 1 (0 - 1) | 1 (0 - 1) | 0.2 (-0.9 - 1.4) | 0.70 |
| Patients with hospital-acquired infections - no. (%) | 7 (10) | 6 (9) | 1.2 (0.4 - 3.7) | 0.79 |
| Barthel Index 3 months - median (IQR) | 100 (100 - 100) | 100 (100 - 100) | 0.1 (-0.8 - 1.0) | 0.79 |
| SF 36 mental 3 months - median (IQR) | 49 (41 - 57) | 47 (40 - 56) | 2.2 (-4.4 - 8.7) | 0.53 |
| SF 36 physical 3 months - median (IQR) | 46 (41 - 52) | 43 (38 - 51) | 2.1 (0.7 - 3.6) | **0.05** |
| 6-minute walking test at hospital discharge (m) – median (IQR) | 237 (112 - 286) | 313 (146 - 340) | -22.0 (-56.5 - 12.4) | 0.51 |

*CI, confidence interval; HR, hazard ratio; ICU, intensive care unit; IQR, interquartile range; no., number of patients; OR, odds ratio; SF 36, short form questionnaire 36. Outcome analyses are purely explorative, as the dataset is not powered to reliably detect differences between the groups.*

***S5: Baseline characteristics and outcomes in patients with selenium baseline levels < 70 µg/l.***

| **Baseline selenium < 70 µg/l (Subgroup III)** | **Selenium**  **(n = 64)** | **Placebo**  **(n = 64)** | **p value** |
| --- | --- | --- | --- |
| **Baseline characteristics** | | |  |
| Age (years) - median (IQR) | 72 (61 - 76) | 72 (63 - 77) | 0.35 |
| Female / Male - no. (%) | 20 (31) / 44 (69) | 17 (27) / 47 (73) | 0.56 |
| Body mass index - median (IQR) | 27 (25 - 30) | 27 (23 - 31) | 0.63 |
| Charlson comorbidity index - median (IQR) | 1 (0 - 2) | 1 (0 - 2) | 0.72 |
| Clinical frailty score - median (IQR) | 3 (2 - 3) | 3 (2 - 3) | 0.24 |
| EuroSCORE II - median (IQR) | 11 (7 - 19) | 9 (6 - 12) | 0.06 |
| Elective / Urgent surgery - no. (%) | 56 (88) / 8 (12) | 60 (94) / 4 (6) | 0.23 |
| Cardiopulmonary bypass (min) - median (IQR) | 141 (117 - 179) | 149 (101 - 196) | 0.95 |
| GFR (ml/min/1.73 m^2^) - median (IQR) | 79 (66 - 94) | 81 (65 - 95) | 0.58 |

*GFR, glomerular filtration rate; IQR, interquartile range; no., number of patients.*

| **Baseline selenium < 70 µg/l (Subgroup III)** | **Selenium (n = 64)** | **Placebo (n = 64)** | **OR, HR or mean differences (95 % CI)** | **p value** |
| --- | --- | --- | --- | --- |
| **Primary outcome** |  |  |  |  |
| Persistent organ dysfunction free days and alive at postoperative day 30 (d) - median (IQR) | 29 (27 - 30) | 29 (27 - 30) | 0.0 (-1.9 - 1.9) | 0.99 |
| **Secondary outcomes** | | | | |
| 30-day mortality - no. (%) | 2 (3) | 5 (8) | 0.4 (0.1 - 2.1) | 0.26 |
| 6-months mortality - no. (%) | 6 (9) | 6 (9) | 1.0 (0.3 - 3.4) | 0.98 |
| Days to discharge alive from ICU (d) - median (IQR) | 2 (1 - 8) | 2 (1 - 6) | 2.0 (-0.7 - 4.7) | 0.27 |
| ICU readmission - no. (%) | 3 (5) | 2 (3) | 1.5 (0.2 - 9.8) | 0.67 |
| Mechanical ventilation (d) - median (IQR) | 1 (0 - 1) | 1 (0 - 1) | -0.3 (-1.4 - 0.8) | 0.59 |
| Patients with hospital-acquired infections - no. (%) | 5 (8) | 7 (11) | 0.7 (0.2 - 2.4) | 0.57 |
| Barthel Index 3 months - median (IQR) | 100 (95 - 100) | 100 (95 - 100) | 1.8 (-2.5 - 6.1) | 0.42 |
| SF 36 mental 3 months - median (IQR) | 46 (36 - 56) | 47 (36 - 57) | 0.7 (-6.2 - 7.5) | 0.85 |
| SF 36 physical 3 months - median (IQR) | 44 (36 - 52) | 43 (37 - 49) | 0.9 (-1.5 - 3.2) | 0.52 |
| 6-minute walking test at hospital discharge (m) – median (IQR) | 236 (100 - 275) | 290 (180 - 347) | -52.8 (-102.0 - -3.6) | 0.40 |

*CI, confidence interval; HR, hazard ratio; ICU, intensive care unit; IQR, interquartile range; no., number of patients; OR, odds ratio; SF 36, short form questionnaire 36. Outcome analyses are purely explorative, as the dataset is not powered to reliably detect differences between the groups.*

***S6: Baseline characteristics and outcomes in patients with glutathione peroxidase*** ***(GPx3) baseline activity < 250 µg/l.***

| **Baseline GPx3 < 250 U/l (Subgroup IV)** | **Selenium**  **(n = 16)** | **Placebo**  **(n = 16)** | **p value** |
| --- | --- | --- | --- |
| **Baseline characteristics** | | |  |
| Age (years) - median (IQR) | 72 (59 - 76) | 75 (69 - 77) | 0.18 |
| Female / Male - no. (%) | 4 (25) / 12 (75) | 4 (25) / 12 (75) | 1.00 |
| Body mass index - median (IQR) | 27 (23 - 30) | 26 (24 - 29) | 0.97 |
| Charlson comorbidity index - median (IQR) | 2 (0 - 3) | 2 (0 - 3) | 0.91 |
| Clinical frailty score - median (IQR) | 3 (3 - 4) | 3 (2 - 4) | 0.59 |
| EuroSCORE II - median (IQR) | 8 (6 - 16) | 9 (6 - 15) | 0.87 |
| Elective / Urgent surgery - no. (%) | 13 (81) / 3 (19) | 14 (87) / 2 (13) | 0.63 |
| Cardiopulmonary bypass (min) - median (IQR) | 164 (151 - 198) | 177 (104 - 210) | 0.94 |
| GFR (ml/min/1.73 m^2^) - median (IQR) | 74 (57 - 88) | 76 (68 - 93) | 0.57 |

*GFR, glomerular filtration rate; GPx3, glutathione peroxidase 3; IQR, interquartile range; no., number of patients.*

| **Baseline GPx3 < 250 U/l (Subgroup IV)** | **Selenium (n = 16)** | **Placebo (n = 16)** | **OR, HR or mean differences (95 % CI)** | **p value** |
| --- | --- | --- | --- | --- |
| **Primary outcome** |  |  |  |  |
| Persistent organ dysfunction free days and alive at postoperative day 30 (d) - median (IQR) | 29 (27 - 30) | 29 (29 - 30) | 0.4 (-5.9 - 6.6) | 0.92 |
| **Secondary outcomes** | | | | |
| 30-day mortality - no. (%) | 1 (6) | 2 (13) | 0.5 (0.0 - 6.6) | 0.57 |
| 6-months mortality - no. (%) | 3 (19) | 3 (19) | 0.9 (0.1 - 6.0) | 0.93 |
| Days to discharge alive from ICU (d) - median (IQR) | 4 (1 - 8) | 3 (1 - 6) | 0.9 (-2.1 - 3.9) | 0.55 |
| ICU readmission - no. (%) | 1 (6) | 0 (0) | n/a | n/a |
| Mechanical ventilation (d) - median (IQR) | 0 (0 - 1) | 0 (0 - 1) | -6.0 (- 6.6 - -5.4) | 0.36 |
| Patients with hospital-acquired infections - no. (%) | 1 (6) | 1 (6) | 1.0 (0.1 - 20.0) | 1.00 |
| Barthel Index 3 months - median (IQR) | 100 (100 - 100) | 100 (100 - 100) | 0.2 (-1.2 - 1.5) | 0.82 |
| SF 36 mental 3 months - median (IQR) | 48 (37 - 56) | 49 (36 - 57) | -0.6 (-6.4 - 5.3) | 0.85 |
| SF 36 physical 3 months - median (IQR) | 46 (41 - 51) | 44 (41 - 49) | 1.0 (-5.8 - 7.7) | 0.77 |
| 6-minute walking test at hospital discharge (m) – median (IQR) | 240 (142 - 430) | 252 (180 - 333) | 28.5 (-117.8 - 174.8) | 0.73 |

*CI, confidence interval; HR, hazard ratio; ICU, intensive care unit; IQR, interquartile range; n/a, not applicable; no., number of patients; OR, odds ratio; SF 36, short form questionnaire 36. Outcome analyses are purely explorative, as the dataset is not powered to reliably detect differences between the groups.*
